# Noninvasive prenatal diagnosis of genetic diseases induced by triplet repeat expansion by linked read haplotyping and Bayesian approach

**DOI:** 10.1038/s41598-022-15307-2

**Published:** 2022-07-06

**Authors:** C. Liautard-Haag, G. Durif, C. VanGoethem, D. Baux, A. Louis, L. Cayrefourcq, M. Lamairia, M. Willems, C. Zordan, V. Dorian, C. Rooryck, C. Goizet, A. Chaussenot, L. Monteil, P. Calvas, C. Miry, R. Favre, E. Le Boette, M. Fradin, A. F. Roux, M. Cossée, M. Koenig, C. Alix-Panabière, C. Guissart, M. C. Vincent

**Affiliations:** 1 Laboratoire de Génétique Moléculaire, Institut Universitaire de Recherche Clinique, Université de Montpellier, CHU Montpellier, 641 Avenue du Doyen Gaston Giraud, 34093 Montpellier Cedex 5, France; 2 IMAG, Université de Montpellier, CNRS, Montpellier, France; 3 PhyMedExp Univ. Montpellier, CNRS, INSERM, Montpellier, France; 4 INM, Institut des Neurosciences de Montpellier, INSERM U1298, Montpellier, France; 5 Laboratory of Rare Human Circulating Cells (LCCRH), University Medical Center of Montpellier, Montpellier, France; 6 Département de Génétique Médicale, Maladies Rares et Médecine Personnalisée, Centre de Référence Anomalies du Développement et Syndromes Malformatifs, Université de Montpellier, CHU de Montpellier, Montpellier, France; 7 Service de Génétique Médicale, Groupe Hospitalier Pellegrin, CHU de Bordeaux, Bordeaux, France; 8 Service de Génétique Médicale, Centre de Référence des Maladies Mitochondriales, Hôpital de l’Archet 2, Nice, France; 9 Service de Génétique Médicale, CHU de Toulouse, Toulouse, France; 10 Department of Maternal Fetal Medicine, Strasbourg University Hospital, Strasbourg, France; 11 Service de Génétique Médicale, Centre Hospitalier de Saint Brieuc, Saint-Brieuc, France

**Keywords:** Biological techniques, Genetics, Molecular biology, Medical research, Molecular medicine

## Abstract

The field of noninvasive prenatal diagnosis (NIPD) has undergone significant progress over the last decade. Direct haplotyping has been successfully applied for NIPD of few single-gene disorders. However, technical issues remain for triplet-repeat expansions. The objective of this study was to develop an NIPD approach for couples at risk of transmitting dynamic mutations. This method includes targeted enrichment for linked-read libraries and targeted maternal plasma DNA sequencing. We also developed an innovative Bayesian procedure to integrate the Hoobari fetal genotyping model for inferring the fetal haplotype and the targeted gene variant status. Our method of directly resolving parental haplotypes through targeted linked-read sequencing was smoothly performed using blood samples from families with Huntington’s disease or myotonic dystrophy type 1. The Bayesian analysis of transmission of parental haplotypes allowed defining the genotype of five fetuses. The predicted variant status of four of these fetuses was in agreement with the invasive prenatal diagnosis findings. Conversely, no conclusive result was obtained for the NIPD of fragile X syndrome. Although improvements should be made to achieve clinically acceptable accuracy, our study shows that linked-read sequencing and parental haplotype phasing can be successfully used for NIPD of triplet-repeat expansion diseases.

**Trial registration:** NCT04698551_date of first registration: 07/01/2021.

## Introduction

Noninvasive prenatal diagnosis (NIPD) of monogenic diseases, based on the analysis of circulating cell-free fetal DNA (cff-DNA) from maternal blood^1–3^, is a safer alternative to invasive prenatal testing methods (amniocentesis and choriocentesis) that entail a significant risk of miscarriage (0.5–1%)^4^. The field of NIPD has undergone significant progress over the last decade. Direct haplotyping has been successfully used for NIPD of a limited range of single-gene disorders: congenital adrenal hyperplasia, Ellis-van Creveld syndrome, hemophilia, Hunter syndrome, cystic fibrosis, β-thalassemia, hemophilia, and Duchenne muscular dystrophy^3,5–7^. However, many tests for NIPD have not been translated into clinical practice because of technical issues related to cff-DNA characteristics and the complexity of the required bioinformatics analyses. Progress in NIPD for monogenic disorders has been much slower compared with the rapid and global implementation of NIPD for aneuploidy, largely owing to the significant commercial drive. Indeed, NIPD for monogenic disorders has attracted less interest because it represents a much smaller market opportunity with a challenging bespoke service, on a patient- or disease-specific basis. Moreover, the used methods and workflows are labor-intensive. Therefore, its implementation remains rare, and most tests are developed and used in research settings, except in the United Kingdom (National Health Service)^8^.

Moreover, not all mutations can be investigated by direct genotyping, particularly triplet-repeat expansion mutations that concern rare and incurable diseases (e.g., myotonic dystrophy type 1, Huntington's disease, fragile X syndrome). The expansion of tandem repeat length across generations is a well-characterized process that results in at least 50 known disorders^9^. These variants are named dynamic mutations. A characteristic of repeat expansion disorders is anticipation. This term describes the appearance of clinical manifestations at an earlier age and/or their increasing severity from one generation to the next. The first expansions, identified on chromosome X in 1991, were CGG repeats in the 5′ untranslated region of the *FMR1* gene (MIM:309550) that are the underlying cause of fragile X syndrome (FXS [MIM: 300624]). It is the most common cause of inherited mental retardation. The full mutation affects 1 in 2500 male individuals who show varying degrees of cognitive and behavioral difficulties, associated with moderate facial dysmorphism. In extreme cases of anticipation, observed in myotonic dystrophy type 1 (DM1) and Huntington’s disease (HD), clinical manifestations can appear in infancy or childhood leading to a fatal outcome in few years. Conversely, the transmitting parent has a milder adult-onset form. DM1 and HD are inherited in an autosomal dominant pattern. DM1 (MIM 160900) is the most frequent adult-onset muscular dystrophy. Its main characteristics are myotonia, progressive muscle weakness and wasting. The underlying mutation is an unstable expansion of CTG repeats in the 3′ untranslated region of the *DMPK1* gene. HD (MIM: 134100) is characterized by irrepressible motor symptoms, cognitive impairment, and psychiatric problems. It is caused by the expansion of a polymorphic trinucleotide (CAG) repeat in exon 1 of the *HTT* gene.

This group of pathologies represents a frequent prenatal diagnosis indication. However, it is challenging to sequence alleles with triplet repeat expansions (CTG, CAG, and CCG) using NGS technologies, especially when the expanded allele size is greater than the length of the short-read sequencing-derived reads, typically between 150 and 300 bp. Additionally, direct haplotype phasing around triplet repeat expansion is required. Recent developments in linked-read sequencing technologies allow overcoming these issues and performing specific haplotyping to more easily determine the haplotypes transmitted to the fetus. A recent study reported the successful application of linked-read direct haplotyping for NIPD in a family at risk of DM1; however, the approach was limited to a single family and depended on an informative single nucleotide polymorphism (SNP; rs635299) linked to the CTG expansion^7,10^, and thus cannot be applied to all families.

Here, we wanted to develop a more general method that can be applied to a wide range of inherited diseases caused by triplet-repeat expansion. We broadened NIPD scope for single-gene disorders using direct linked-read haplotyping. We modified the approach proposed by Hui et al.^5^ and applied direct phasing for NIPD in families at risk of transmitting triplet-repeat expansion mutations to their fetus. We focused on three genetic diseases: HD, DM1, and FXS.

Our study describes the necessary steps for targeted noninvasive fetal genotyping, including targeted enrichment for linked-read libraries and targeted maternal plasma DNA sequencing. Then, a Bayesian approach was used to infer the fetal genotype and identify each parental allele transmitted to the fetus. This approach incorporates information on direct parental haplotype phasing, fetal DNA fraction, and sequencing data from maternal plasma DNA samples. Our Bayesian method for NIPD of triplet-repeat diseases extends the methodology introduced in the Hoobari software, the first tool for the genome-wide detection of fetal point mutations that has recently become available^11^. We propose an innovative Bayesian procedure to integrate the Hoobari fetal genotyping model in order to infer the parental origin (i.e., the transmitted haplotype) of each fetal allele at each locus of a target chromosome region. Our method is independent of the inheritance mode and parental origin. This new and easily adaptable method allows the NIPD of theoretically any monogenetic disorder in families at risk of transmission.

## Methods

### Patients and samples

The study participants were couples at risk of transmitting one of the three target triplet-repeat diseases to their fetus. Genomic DNA from both parents and plasma DNA samples from the pregnant women were from a sample collection for research purposes that was approved by a Research Ethics Committee (Personal Protection Committee, CPP 2017-A00232-51/3 on 18/04/2017, Modif. Subst. 19-180/ref. CPP 17-CHUM-01 and Agence Nationale de Securite du Medicament ANSM: n°ID-RCB:2017-A00232-51, 25/04/2017). Couples at risk of having a fetus with a triplet-repeat disease provided their written informed consent. All study methods were carried out in accordance with the relevant guidelines and regulations.

In total, 14 couples were included in this study (n = 5 at risk of transmitting HD, n = 7 at risk of transmitting DM1, and n = 2 at risk of transmitting FXS (Table 1)).

**Table 1:** Data of studied families.

Family no	Disease	Gene	Affected/carrier parent	Gestational age (weeks)
1	Myotonic dystrophy	DMPK	Mother	9
2	Myotonic dystrophy	DMPK	Father	11
3	Myotonic dystrophy	DMPK	Father	11
4	Myotonic dystrophy	DMPK	Father	11
5	Myotonic dystrophy	DMPK	Mother	10
6	Myotonic dystrophy	DMPK	Father	24
7	Myotonic dystrophy	DMPK	Mother	11
8	Fragile X syndrome	FMR1	Mother	21
9	Fragile X syndrome	FMR1	Mother	12
10	Huntington	HTT	Mother	10
11	Huntington	HTT	Father	11
12	Huntington	HTT	Father	11
13	Huntington	HTT	Father	11
14	Huntington	HTT	Mother	12

**Figure 1:**
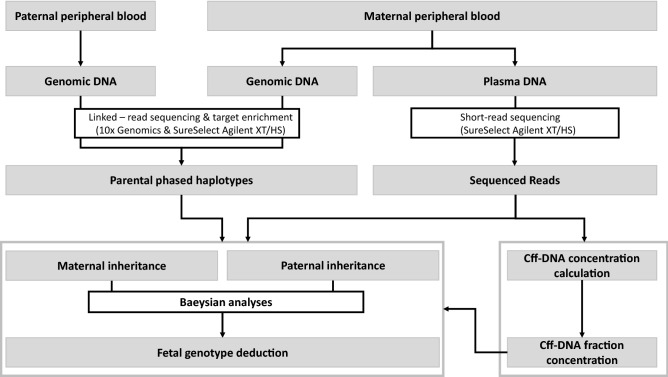
Flow chart of the overall strategy for noninvasive prenatal diagnosis of triplet-repeat expansions.

Pregnant women underwent prenatal diagnosis (gold standard) by amniocentesis or choriocentesis between week 9 and 24 of gestation after blood sampling for NIPD. Couples were included in the study at one of the participating medical genetic centers during a genetic counseling consultation. During this visit, blood samples were collected in Streck (3 × 10 mL) and EDTA (1 × 5 mL) tubes from the pregnant women, and in EDTA tubes (1 × 5 mL) from the future fathers. Blood samples were processed within 24 h (Streck tubes) or within 7 days (EDTA tubes). Blood samples collected in Streck tubes were used for cff-DNA isolation and analysis. Figure 1 illustrates the flowchart of the overall study strategy.

### DNA extraction

Using 30 ml of blood from Streck tubes, plasma was separated from the cellular fraction by centrifugation at 1600*g* and then at 14,000*g* for 10 min/each. Plasma samples were frozen at − 80 °C until further processing. Cell-free DNA (cf-DNA) was extracted from 4 ml of thawed plasma samples using the QIAsymphony DSP Circulating DNA Kit (Qiagen, Courtaboeuf, France) and the QIASymphony instrument, according to the manufacturer’s protocol. Concentrated and purified cf-DNA was eluted in a final volume of 60 μl of AVE buffer.

Parental genomic DNA was extracted from whole blood collected in EDTA tubes with the FlexiGen DNA kit (Qiagen) according to the manufacturer’s protocol for genotyping.

### Assay design/target region capture

Windows of approximately 200 kb were defined around each focal triplet expansion, based on the hg19 human genome assemblage. The GnomAD 2.1.1 database was used to identify, within these windows, common polymorphic sites known in the human genome worldwide. Among all the known polymorphic sites, the one for which the minority allele had a frequency ≥ 0.1 for the DM1 and ≥ 0.07 for the FXS and HD regions were selected because their harbor a lower polymorphism level. Their position was given to the Agilent technical support team to design RNA capture probes around most of these targeted SNPs. Whenever possible, two overlapping probes (2× tilling) of 120 bp were designed around each SNP. A region of 847 bp on chr4, 3174 bp on chr19, and 2510 bp on chrX around the targeted triplet expansion was covered by 5× tilling. The probes covered a total of 172,432 bp (chr4: 56,442 bp—660 probes, chr19: 55,830 bp—662 probes, and chrX: 60,160 bp—701 probes).

### Library preparation/sequencing

Library preparation was partly performed separately for the parental genomic DNA and plasma DNA. The study region was selected from the whole genome DNA by capture with the SureSelect XT HS Reagent Kit of the Agilent kit for all three samples of each family.

#### Libraries of genomic DNA

The average fragment size of parental DNA was controlled on a Genome Tape (Tape Station, Agilent). If the sample had a DNA integrity number (DIN) ≥ 9, the sample was directly used for library preparation. If the DIN was < 9, the average fragment size was < 50 kb, and DNA fragments were sorted on BluePippin and with "High-Pass DNA Size Selection" Tape (Sage Science) at a threshold of 30 kb. High molecular weight DNA samples from both parents were then used to construct an Illumina-compatible library with the recommended protocol for 10× Genomics "Linked reads" (Chromium™ Genome Reagent Kits v.2., 10× Genomics, Pleasanton, CA). Once the 10× Genomics library was constructed, target enrichment was performed using the Agilent "SureSelectXT/HS Low Input" custom kit (including the targeted genes) according to the manufacturer’s recommendations.

#### Libraries of maternal plasma cf-DNA

Libraries of cf-DNA were prepared using the "SureSelect XT HS/XT Low Input” Target Enrichment System for Illumina Paired-End Multiplexed Sequencing Library" following the provider’s (Agilent) protocol, except for sonication that was not done. Indeed, cf-DNA is already fragmented to a mean size of approximately 140 bp.

#### Pooling and sequencing

Following hybridization and successful amplification, post-capture libraries were evaluated on an Agilent 4200 TapeStation system (Agilent Technologies) using High Sensitivity D1000 ScreenTape. All libraries were pooled in equimolar status based on the TapeStation results, except for maternal plasma cf-DNA libraries that were 1.2 times more concentrated to achieve better sequencing coverage. Pools were sequenced (150 paired-end reads) on an Illumina NextSeq500 in Mid Output Flow Cell Cartridge v.2.5 output mode (v2 or v2.5, Illumina). All prepared libraries were sequenced in a single run.

### Data analysis/custom-made bioinformatics pipeline

#### Sequencing data

**Figure 2:**
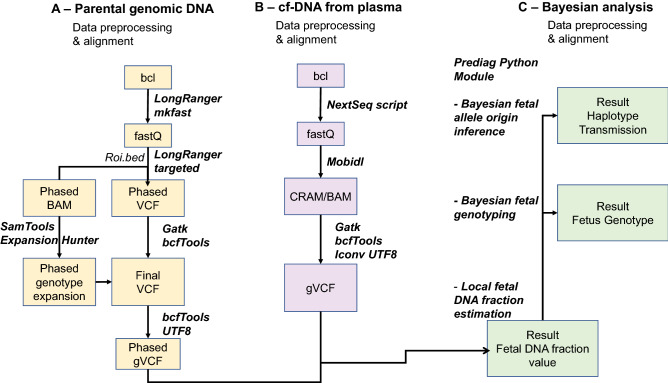
Different steps of the bioinformatic pipelines used in our study. The third step is a modified Hoobari pipeline for noninvasive prenatal genotyping.

Bioinformatic analyses of the sequencing data were partly performed separately for plasma cf-DNA libraries and 10× gDNA libraries to generate the genomic variant calling files (gVCF) files necessary for fetal allele origin inference (Fig. 2).

#### Plasma cf-DNA variant calling

Illumina raw data for plasma cf-DNA samples were demultiplexed using Illumina bcl2fastq. Fastq files were processed into standard VCFs by the MobiDL pipeline (https://github.com/mobidic/MobiDL, v1.1).

#### Parental genomic DNA variant calling and phasing

Parental genomic DNA libraries constructed with the 10× Genomics technology were processed from bcl to VCF using the Long Ranger software from 10X Genomics (software Loupe 2.1.1

https://www.10xgenomics.com/products/loupe-browser, 10× Genomics).

#### gVCF generation and compilation for each family

An in-house script generated the gVCF and compiled the genomic data for each family. A VCF and a BAM/CRAM were generated for each sample. Low-quality mapping reads (< 20) and secondary alignment were discarded, and poly-A and -T were filtered out for each sample. All samples from a given family (mother, father, and mother plasma) were treated together. First, starting from the alignment files (BAM/CRAM), a gVCF was generated to include all alternated alleles of the family, heterozygous or homozygous for at least one sample. A SNP list was then produced with all the positions that were polymorphic in at least one of the three family samples.

#### Analysis of the triplet-repeat expansion

The triplet-repeat expansion could not be genotyped with traditional pipelines. Expansion Hunter (https://academic.oup.com/bioinformatics/article/35/22/4754/5499079, ExpansionHunter-v4.0.1-linux_x86_64.tar.gz) was used to correctly genotype this mutation type when possible. First, the genomic DNA from each parent was analyzed to identify heterozygous SNPs. Each BAM was phased by Longranger and then split into two files with a custom script to obtain a BAM file that contained only the reads of a single haplotype. Thus, for each subject, two BAM files were obtained that were associated with at least one of the two chromosome strands. Then, each BAM file was genotyped again with Expansion Hunter to obtain the number of repeats linked to each allele of the chromosome (i.e., phased with the other discovered SNPs). In some difficult cases, when triplet repeat expansions were very long, the script allowed phasing the healthy allele and by deduction, identifying the haplotype that carried the affected allele.

#### Fetal fraction

The fetal fraction^12,13^ quantifies the proportion of cff-DNA that can be found among the cf-DNA in the mother’s plasma. It was estimated based on the maternal and paternal genotypes and the genotype inferred from the plasma cf-DNA, together with the corresponding allelic depths^14–16^. The number of reads that covered each allele in the plasma cf-DNA over all the SNPs found in the study region was used to infer the fetal DNA frequency in the plasma cf-DNA (for more details, see supplementary materials).

#### Fetal allele origin inference

A Bayesian approach was developed to infer the fetal allele origin from the parental phased haplotypes at each targeted locus. This Bayesian approach was implemented as a Python package called prediag (version 1.0.1), with a command line interface (CLI). The source code can be found in a dedicated repository (https://github.com/gdurif/nipd). Our aim was to identify, for a given genomic region, the parental origin of the genetic material inherited by the fetus without using a proband. To do so, only haplotype data from both parents and genotype data from the maternal plasma cf-DNA (i.e., a mix of maternal and fetal DNA) were used.

Our Bayesian framework estimates the joint posterior probability

$$P\left({O}_{1},\ldots ,{O}_{\ell},\ldots ,{O}_{L}|{\text{data}}\right)$$

of the parental allele origin ${O}_{\ell}$ in the fetus for all loci $\ell=1,\ldots,L$ in the region of interest. Because of the dependency between consecutive loci concerning the parental allele inheritance, which is only broken in case of a rare recombination event in one or both parents (in the order of 100 kb considering the region width), the joint posterior cannot be decomposed as the product of marginal posteriors at each locus (as in the fetal genotyping model). To overcome this issue, a Markov chain Monte Carlo (MCMC) algorithm was used^14^, specifically a Gibbs sampler^15,17^, to estimate the full posterior and then infer the allele origin. Our MCMC procedure is based on the conditional marginal posterior

$$P\left({O}_{\ell}|{\text{data}},{O}_{\ell-1}\right)\sim P\left(\text{data at locus }\ell|{O}_{\ell}\right)\times P\left({O}_{\ell}|{O}_{\ell-1}\right)$$

where (i) “data” refers to the parental haplotypes and the cf-DNA genotype at the considered locus; (ii) the data likelihood $P \left(\text{data at locus }\ell|{O}_{\ell}\right)$ is computed using the Hoobari fetal genotype model^11^ that has been modified to allow discriminating maternal and paternal alleles in fetal heterozygous loci; and (iii) the transition probability $P\left({O}_{\ell}|{O}_{\ell-1}\right)$ between consecutive loci depends on the distance between the considered loci, the recombination rate, and the estimated probability of phasing errors (in function of the phasing procedure) in the parental haplotypes (which could also explain a switch of the parental allele origin in the fetus).

For more details about our Bayesian method, see supplementary materials.

### Ethics declaration

The study was approved by the local ethics committee (Personal Protection Committee, CPP 2017-A00232-51/3 on 18/04/2017, Modif. Subst. 19-180/ref. CPP 17-CHUM-01 and Agence Nationale de Securite du Medicament ANSM: n°ID-RCB:2017-A00232-51, 25/04/2017).

All individuals signed a written consent prior to genetic analysis.

## Results

### Sequencing data

The targeted linked-read sequencing of 28 parental genomic DNA samples showed relatively consistent coverage throughout the targeted region, with a mean coverage of 141 reads (see Table S1, supplementary data). Coverage was significantly lower for the parent genotype than for the plasma genotype, as expected based on the higher plasma cf-DNA library concentration during sequencing (parents: 95X, vs plasma: 212X).

The mean number of polymorphic sites (SNPs) found in a given parent was 240 SNPs per parent for the *DMPK* gene and 167 SNPs for the *HTT* gene. No data from the two families at risk of transmitting FXS was obtained due to misalignment of the reads containing the CGG expansion of the *FMR1* gene. Therefore, the expansion could not be genotyped, and the phasing analyses could not be performed.

### Parental haplotype phasing

Among the other twelve families, at least a partial phasing of both parental haplotypes could be established in 23 parents (82%, Table 2), with a mean bloc size of 128,699 bp (min: 1259 bp, max: 216,806 bp) (Table S1, supplementary data). However, at the family level, only regions > 50 kb could be phased in both parents in 6/12 families. These six families had many informative SNPs (i.e., a significant number of positions for which at least one of the parents was not homozygous for the reference allele). These informative SNPs, hereafter called “shared SNPs”, were genotyped in both parents and in the maternal plasma sample. The mean number of shared SNPs that could be used to deduce the haplotype transmitted by each parent was 68 (0 to 188 SNPs). The number of shared sites for each family was strongly correlated with the phasing quality in the less well phased parental haplotype.

**Table 2:** Results of the bioinformatic analysis with the deduced NIPD genotypes.

ID	Affected/carrier parent	Phased block size (bp)	Haplotype affected/carrier	Shared positions^a^	Estimated foetal fraction	Inherited parental allele	NIPD outcome	Invasive PND outcome	NIPD vs PND comparaison
1	Mother	10,412	ND	0	ND	ND	ND	Affected	Non conclusive
2	Father	198,069	Hap1	185	17%	Hap2	Not affected	Not affected	In agreement
3	Father	12,882	ND	10	9%	ND	ND	Affected	Non conclusive
4	Father	119,004	Hap2	127	19%	Hap2	Affected	Affected	In agreement
5	Mother	34,019	ND	0	ND	ND	ND	Affected	Non conclusive
6	Father	198,069	ND	0	ND	ND	ND	Not affected	Non conclusive
7	Mother	197,608	Hap2	186	17%	Hap2	Affected	Not affected	Discordant
10	Mother	0	ND	0	ND	ND	ND	Not affected	Non conclusive
11	Father	216,324	Hap2	188	13%	Hap2	Affected	Affected	In agreement
12	Father	59,888	ND	26	14%	ND	ND	Not affected	Non conclusive
13	Father	165,060	Hap2	95	12%	ND	ND	Not affected	Non conclusive
14	Mother	215,750	Hap2	4	26%	Hap2	Affected	Affected	In agreement

^a^Number of positions presenting at least one copy of an alternate allele compared to the reference genome in at least one of the parents and for which all three samples could be genotyped.

### Fetal fraction

The fetal fraction can be calculated only when the genotype of all samples for several genomic positions can be obtained from a given family. In this study, it could be estimated for 8/12 families with a mean value of 16% (min 9%; max: 26%, Table 2).

### Fetal allele origin inference

For these eight pregnancies, the Bayesian analysis of the parental haplotype transmission allowed us to infer the genotype and allele origin in five fetuses (36% of the studied pregnancies). Four (80%) were in agreement with the results obtained with the gold standard test (amniocentesis/choriocentesis): three fetuses carried the disease-causing mutation and one fetus did not (Table 2).

## Discussion

A conclusive result could be obtained for 5 of the 14 studied families. Our conclusion about the haplotype transmitted by the affected parent was identical to the results given by amniocentesis in 80% (n = 4/5) of fetuses. However, for one family (20%), no accurate NIPD result was obtained. We could not explain this negative result specifically. We checked and did not detect any sample identification error, although this hypothesis could never be completely excluded. A second analysis should have been performed to confirm or exclude this discrepancy. However, due to our protocol design, it was not possible (i) to perform again the analysis due to the limited plasma quantity collected from the pregnant mother, and (ii) to request new samples to exclude a problem of identity at the time of sample collection and/or anonymization.

We managed haplotype phasing of sequences with a mean length of 129 kb around the expansions in 23/28 parents. The phase block size across the target region was smaller compared with the studies by Chen et al.^18^ and Lee et al.^10^ who reported phase blocks with of a mean size of 741 and 632 kb, respectively. Their higher phasing success was mainly due to the larger width of the targeted sequencing region (657 kb and 3.2 Mb, respectively). We opted for targeted sequencing of the most polymorphic sites in a 200 kb region around the expansion to develop an affordable test for clinical practice. Similarly, Jang et al.^19^ used a smaller phase block (42 kb) and obtained phasing results that were adequate for all subsequent analyses. They explained that if linked-read sequencing can be applied in the framework of a limited targeted approach without the need for a large capture probe design, NIPD application could be broadened in clinical practice due to the reduced costs.

Unfortunately, in our experimental conditions, the phasing results were not as good as expected because of the short targeted and discontinued sequencing regions. The mean phased block size of the least well-phased parental haplotype was 138 kb among the families for whom we could reach a conclusion, but only 39 kb among the families for whom we could not reach a conclusion. The full study region was perfectly phased in 12/23 parents, whereas < 50 kb phased blocks were obtained for the other parents. This clearly shows the importance of the phased region size. In our families, optimization of the phasing analysis by linked-read sequencing using the 10X Genomics technology would have required testing a new library design and the sequencing of a larger continuous region. However, the commercialization of the Chromium™ Genome Reagent Kit was stopped when we obtained the first results, thus precluding any further optimization. To identify what key parameters should be improved to increase significantly our method performance, we carried out statistical analyses (see supplementary data). They showed that the our method efficiency was influenced by different, closely linked parameters. Specifically, the sequencing data depth and the SNP number strongly influenced the success of the diagnosis by affecting the parental haplotype phasing and fetal DNA fraction estimation. Without these two elements, the pipeline could not continue the data processing to determine the fetus genotype. The low number of shared sites (i.e., few informative SNPs) could be explained by a lack of informativity in the targeted region. Moreover, the low number of shared sites among parents was strongly dependent on the size of the successfully phased region around the expansion for both parents.

Regarding the nine families with unconclusive results, we could not run our inference method because the necessary input data could not be extracted or because they were too incomplete to obtain a sufficiently accurate result. Similarly, we could not genotype the triplet-repeat in the two families at risk of transmitting FSX, thus excluding them from the subsequent analyses. In these two families, the expansion could not be sequenced due to the too short reads, and thus could not be phased. For seven families, we did not obtain any result due to the lack of informative SNPs in the targeted region between parents and maternal plasma. Nevertheless, we think that the weak efficiency of our method is mainly due to molecular biology technical issues that will be surely and rapidly overcome due to the continuous improvement of long-read sequencing and/or new linked-read sequencing technologies.

### Advantages and limits of our approaches vs already published approaches

Our approach allows the reconstruction of the parental haplotypes by linked-read direct haplotyping even when the proband’s genotype is unavailable.

The Bayesian approach used to determine the genotype and allele origin in the fetus is independent of the inheritance mode and parental origin of the disease.

The proposed tools can help biologists in accessing NIPD data analysis. Therefore, our approach could be applied to any rare monogenic disease.

Our results are encouraging in view of the rare published data on NIPD for dynamic mutations based on the analysis of circulating DNA: three indirect studies of the transmission of the paternal morbid allele^20,21^ and two direct studies of two families at risk of maternal transmission^10,22^. The first three papers describe an indirect method that does not allow NIPD if the carrier parent is the mother. Our test has overcome this limitation of multiplex PCR-based tests that cannot predict which maternal allele is inherited by the fetus. The direct approach for NIPD of DM1 is limited to only one family and depends on an informative SNP (rs635299) linked to the CTG expansion^7,10^, which cannot be used for all families. Indeed, we found that in our families, rs635299 was informative in only 25% of cases. The latest study on NIPD for single gene disorders^22^ concerned testing for FXS in a single family. A conclusive result was obtained from the analysis of three fetal DNA molecules, two of which carried recombinant events. This approach, based on long-read sequencing with the Oxford Nanopore Technology needs to be confirmed by assessing other families. The maternal haplotypes, required for this approach, were deduced from the mother and proband’s genotype data. Our approach can distinguish each parental haplotype, while phasing it with the pathogenic variant involved in the disease, without the need of proband’s DNA. Therefore, our NIPD approach can be proposed regardless of the monogenic disease type, transmission mode, genetic anomaly nature and availability (or not) of proband’s DNA.

Our method needs to be improved to achieve clinically acceptable accuracy and overcome the following issues:

- The phasing quality of the parental genomic DNA, which was very low for some couples.
- The difficulty of sequencing the triplet-repeat regions when using short-read sequencing: the normal-sized allele is relatively well defined and this allows by deduction to identify the phase associated with the morbid allele, which is only partially sequenced (the obtained size of the pathogenic expansions was < 50 repeats, whereas in our population, subjects at risk of transmitting DM1 carried an expansion of several hundred repeats).
- The number of informative SNPs for phasing: we designed our library to cover approximately 300 useful SNPs per family, and the analyses gave us only 68 SNPs (mean value). This difference can be linked to the actual frequency of these SNPs in the studied population and/or to the use of too stringent quality criteria in our bioinformatic pipelines.

## Conclusions

Our study demonstrates the potential contribution of our approach to NIPD of triplet-repeat expansion disorders, such as DM1 and HD. However, this approach requires additional technical improvements to achieve clinically acceptable accuracy for NIPD of many different single-gene disorders.

Our indirect method based on the haplotyping of parental genomic DNA without index cases by linked-read sequencing (10× Genomics) associated with the targeted sequencing of DNA from maternal plasma represents a possible alternative to the current prenatal diagnosis tools. We obtained convincing results for four pregnancies at risk of transmission of a disease-causing triplet expansion (MD1 and HD). However, it did not allow the analysis of dynamic mutations rich in CG (CGG repeats of large size in FXS). The proposed approach still faces technical challenges and requires optimizing complex bioinformatics analyses. Long-read sequencing is currently a very dynamic field. We can expect very soon the development of a new technique that might be implemented in our protocol to achieve high-quality haplotype phasing in any family.

## Supplementary Information

Supplementary Legends.

Supplementary Information 2.

Supplementary Information 3.

Supplementary Table S1.

## Data Availability

The datasets generated and analyzed in this study are not publicly available because they are health data in the field of clinical genetics and thus they are covered by specific national regulations in terms of confidential and security. Part of the data are available from the corresponding author on reasonable request.
