# Supplementary material for: Noninvasive prenatal diagnosis of genetic diseases induced by triplet repeat expansion by linked read haplotyping and Bayesian approach
Source: Sci Rep. 2022 Jul 6;12:11423. doi: 10.1038/s41598-022-15307-2 (PMC9259573; doi:10.1038/s41598-022-15307-2)
Supplement: Supplementary file 1 — Supplementary Legends. [file 41598_2022_15307_MOESM1_ESM.pdf]

**Figures Captions**

**Figure 1:** Flow chart of the overall strategy for noninvasive prenatal diagnosis of triple-repeat expansions.

**Figure 2:** Different steps of the bioinformatic pipelines used in our study. The third step is a modified Hoobari pipeline for noninvasive prenatal genotyping.

**Supplementary material**

**Supplemental Table 1S:** Raw NGS data

**Supplemental materials:** Details of our Bayesian approach

Supplemental Data S3: Statistical analyses

**Additional information:**

The source code for our method is available in this repository: https://github.com/gdurif/nipd
