# Supplementary material for: Noninvasive prenatal diagnosis of genetic diseases induced by triplet repeat expansion by linked read haplotyping and Bayesian approach
Source: Sci Rep. 2022 Jul 6;12:11423. doi: 10.1038/s41598-022-15307-2 (PMC9259573; doi:10.1038/s41598-022-15307-2)
Supplement: Supplementary file 2 — Supplementary Information 2. [file 41598_2022_15307_MOESM2_ESM.pdf]

Non-invasive prenatal diagnostic of genetic diseases induced by triplet repeats expansion by linked reads haplotyping and Bayesian approach

Supplementary materials 2: methodology

June 14, 2022

Liautard-Haag C.[1], Durif G.[2], Van Goethem C.[1,3], Baux D.[1,4], Louis A.[1], Cayrefourcq L.[5], Lamairia M.[1], Willems M.[6], Zordan C.[7], Dorian V.[7], Rooryck C.[7], Goizet C.[8], Chaussenot A.[8], Monteil L.[9], Calvas P.[9], Miry C.[10], Favre R.[10], Le Boette E.[11], Fradin M.[11], Roux AF.[1,4], Cossée M.[1,3], Koenig M.[1,3], Panabière C.[3], Guissart C.[1], Vincent MC.[1,3]

[1] Laboratoire de Génétique Moléculaire, Institut Universitaire de Recherche Clinique, Université de Montpellier, CHU Montpellier, Montpellier, France;
[2] IMAG, Université de Montpellier, CNRS, Montpellier, France;
[3] PhyMedExp Univ. Montpellier, CNRS, INSERM, Montpellier, France;
[4] INM, Institut des neurosciences de Montpellier, INSERM U1298, Montpellier, France;
[5] Laboratory of Rare Human Circulating Cells (LCCRH), University Medical Center of Montpellier, Montpellier, France;
[6] Département de Génétique Médicale, Maladies Rares et Médecine Personnalisée, Centre de Référence Anomalies du Développement et Syndromes Malformatifs, Université de Montpellier, CHU de Montpellier, France;
[7] Service de Génétique Médicale, Groupe Hospitalier Pellegrin, CHU de Bordeaux, Bordeaux, France;
[8] Service de Génétique Médicale, Centre de Référence des Maladies Mitochondriales, Hôpital de l'Archet 2, Nice, France;
[9] Service de Génétique Médicale, CHU de Toulouse, Toulouse, France;
[10] Department of Maternal Fetal Medicine, Strasbourg University Hospital, Strasbourg, France;
[11] Service de Génétique Médicale, Centre Hospitalier de Saint Brieuc, Saint-Brieuc, France.

Contents

S2.1 Computational Tools **3**
S2.1.1 Pipeline implementation and software 3
S2.1.2 Genotype and haplotype 4
S2.1.3 cfDNA genotype correction and filtering 5
S2.1.4 Bayesian approach for noninvasive fetal allele origin inference 5
S2.1.4.1 Fetal fraction estimation 6
S2.1.4.2 Fetal genotype model 6
S2.1.4.3 Fetal allele origin inference 8

S2.A1 Fetal fraction estimation **11**
S2.A1.1 Local fetal fraction 12
S2.A1.2 Smoothing 16

S2.A2 Fetal genotype model **16**
S2.A2.1 Fetal genotype prior 16
S2.A2.2 Data likelihood 16

S2.A3 Fetal allele origin inference **19**
S2.A3.1 Reminder about Gibbs sampling 23

S2.1 Computational Tools

Here is a presentation of the computational tools used in our work, and especially a description of our Bayesian approach for noninvasive fetal allele origin inference, as well as specific data preprocessing. Our pipeline is based on different steps, including fetal fraction estimation, fetal genotype inference, and eventually our main contribution: an innovative method to infer the fetal allele origin from parental phased haplotypes.

Our approach requires a genotyping of both parents DNA, and a genotyping of the *circulating free DNA* (cfDNA) from the maternal plasma, which is a combination of both cell-free maternal DNA and *cell-free fetal DNA* (cffDNA). Thus, the cfDNA genotype is a combination of both the maternal and the fetal genotype.

See section S2.1.1 for more details about our method implementation and availability. Section S2.1.2 introduces some notations about genotypes and haplotypes. Section S2.1.3 details data preprocessing. Eventually, section S2.1.4 presents our Bayesian approach.

Additional informations are attached in a specific appendix for this document (referenced wherever needed in the different sections).

S2.1.1 Pipeline implementation and software

The source code for our method is available in this repository https://github.com/gdurif/nipd as a Python package (called `prediag`), along with a command line interface (CLI).
Here is an outline of the pipeline implemented in our software:

1. Fetal fraction estimation
2. Fetal genotype Bayesian inference
3. Initialize fetal allele origin with the following heuristic
    - use inferred fetal genotype to infer fetal allele origin at parental heterozygous (not ambiguous) locus
    - use previously inferred fetal allele (at parental heterozygous locus) to infer fetal allele origin at parental homozygous (ambiguous) locus by a vote in the locus neighborhood

4. Bayesian inference of fetal allele origin in fetus with Gibbs sampling

See the following for more details about each step.

S2.1.2 Genotype and haplotype

Here we introduce some notations for genotypes and haplotypes that will be used later. We focus on a diploid model, i.e. with a pair of homologous copies for each chromosome, excluding X-Y chromosomes (for the moment) and all chromosomal abnormality in karyotype

Genotype. At a given locus presenting a *Single Nucleotide Polymorphism* (or SNP), the *genotype* refers to the combination of alleles carried by homologous chromosomes. In diploid species as humans, the genotype for a given locus will be noted

$$x/y \in \{\, 0/0,\ 0/1\, 1/1 \,\},$$

where $x, y \in \{0, 1\}$ identify the two alleles present at the locus. Generally, 0 refers to the ancestral allele, and 1 refers to the derived or alternative allele. Locus with genotype 0/0 or 1/1 are called homozygous. For heterozygous locus, i.e. with alleles 0 and 1, the genotype is noted (by convention) 0/1 (which is equivalent to 1/0, but the former notation is not used).

We also removed all the remaining positions with three or more alleles, which were generally genotyping errors left, i.e. we consider SNPs with two possible alleles at most (0 or 1). Considering the size of the human genome and the mutation rate, the probability of two mutations arising at the exact same locus at different moment of the human evolution is very small. Thus, the proportion of SNPs with more than two possible alleles is considered negligible.

Haplotype. On contrary to genotypes, phased *haplotypes* (i.e. haploid genotypes) allow to decipher which allele is carried by which chromosome (in a pair of homologous chromosomes) at a given locus. Deriving the haplotypes from a given genotype is called *phasing* or *haplotype estimation* (see Huang, Tu, and Lu, 2017 or Choi et al., 2018 for a review).

Phased haplotypes for a given locus are noted

$$a|b \in \{\, 0|0,\ 0|1,\ 1|0,\ 1|1 \,\},$$

where $a \in \{0,1\}$ corresponds to the allele carried by the 1st haplotype/ chromosome (noted 1) and $b \in \{0,1\}$ corresponds to the allele carried by the 2nd haplotype/chromosome (noted 2) among the pair of homologous chromosomes. It should be noted that, in diploid species as humans, the pair of homologous haplotypes noted $a|b$ for a given locus is generally referred to as the haplotype (singular) for this given locus .

S2.1.3 cfDNA genotype correction and filtering

cfDNA genotypes were corrected after the variant calling analysis, based on the allelic depth. At each locus, we only considered alleles for which the corresponding allelic depth (i.e. the number of reads mapped on the locus carrying the considered allele) was higher than both an absolute threshold (2 reads) and a relative threshold (1% of the coverage at the locus).

cfDNA locus were filtered based on a minimum coverage criterion of 50 mapped reads.

S2.1.4 Bayesian approach for noninvasive fetal allele origin inference

We propose a *Bayesian approach* (see Bolstad and Curran, 2016, for an introduction about Bayesian statistics) to *infer the fetal allele origin* in the parental phased haplotypes at the locus scale. In particular, at each locus, we aim not only to infer the fetal genotype using both parental genotypes and the cfDNA genotype, like in the Hoobari approach (Rabinowitz et al., 2019), but also to determine which allele from which chromosome was given by each parent to the fetus.

Extending Hoobari model to infer fetal genotype using parental genotypes and cfDNA genotype (Rabinowitz et al., 2019), our method is able to infer which allele was given by each parent to the fetus at each locus in a target region. To do so, we use a *Markov chain Monte Carlo* (MCMC) algorithm (see Andrieu et al., 2003, for an introduction), and specifically a *Gibbs sampler* (S. Geman and D. Geman, 1984), to account for the dependency between consecutive locus regarding parental allele inheritance.

To implement our approach, an estimation of the fetal fraction in the cfDNA sample is required (c.f. section S2.1.4.1). Then, we integrate a specific model on the fetal genotype (c.f. section S2.1.4.2) to infer the fetal allele origin

(c.f. section S2.1.4.3).

S2.1.4.1 Fetal fraction estimation

The fetal fraction (see Hui and Bianchi, 2020, for a review) is the proportion of genetic material (i.e. reads) that originate from the fetus in the cfDNA sample. It is a crucial parameter in our model and should be estimated carefully (c.f. Peng and Jiang, 2017). In our approach, fetal fraction is first estimated at a locus resolution (i.e. for each SNP where it is possible) and then smoothed along the genome by averaging the local estimates on sliding windows (of 100kb width).

At the locus scale, the fetal fraction was estimated based on both parents and cfDNA genotype, and corresponding allelic depths (Lo et al., 2010; Chan and Jiang, 2015). In particular, for certain combinations of parental genotypes, it is possible to quantify the theoretical contributions of maternal and fetal genotypes to the cfDNA allelic depth, and then estimate the corresponding fetal fraction.

The per-locus fetal fraction estimation accuracy depends on the per-locus coverage (the higher coverage the better estimation). To correct for this coverage effect (and get an estimate for the locus where the parental and cfDNA genotypes are not informative to determine the fetal fraction), a smoothing is done based on a weighted averaging of fetal fraction estimations in a sliding window of size 100kb centered in each locus. The average weights are proportional to the locus coverage to reduce the effect of the low coverage locus where the accuracy of the fetal fraction estimation is lower.

More details regarding the fetal fraction estimation can be found in appendix section S2.A1.

S2.1.4.2 Fetal genotype model

The fetal genotype model in our approach is based on Hoobari fetal genotype model (Rabinowitz et al., 2019) with a slight modification allowing to infer the fetal genotype and from which parent (i.e. the mother or the father) fetus inherited the derived allele for heterozygous locus.

More details regarding the fetal genotype model can be found in appendix section S2.A2.

Fetal genotype posterior. For a given locus, the posterior on the fetal genotype G is given by:

$$P(G|\text{data}) = \frac{P(\text{data}|G)\,P(G)}{\sum_g P(\text{data}|G_g)\,P(G_g)} \tag{S2.1}$$

where $\{G_g\}_g$ are all possible fetal genotypes.

Then, we introduce a modification to the genotype standard notation: a fetal genotype will be encoded a/b where $a \in \{0, 1, \ldots\}$ and $b \in \{0, 1, \ldots\}$ identify the maternal and paternal alleles respectively (i.e. the allele inherited from the mother and from the father respectively). The interest of this notation is to account for the mutated allele origin (from the mother or the father) when the fetus is heterozygous at a locus. For instance, with the standard encoding, an heterozygous locus will be encoded 0/1 and the parental origin of both alleles cannot be identified. Here, with our notation, an heterozygous locus can be encoded 1/0 or 0/1 depending if the derived allele was inherited from the mother or the father respectively.

It should be noted that this notation convention is only useful to derive the posterior in the model, and was solely introduced for this purpose. We stress out that altough it seems quite similar to fetal haplotype phasing, since we process each locus independently (which is not suitable for haplotype phasing), we prefer to use this non standard genotype notation instead of the haplotype notation.

Computing the posterior requires computing the data likelihood $P(\text{data}|G)$ and the genotype prior $P(G)$ which are explicit (c.f. below). Thus, the posterior can be computed at the locus scale, and the fetal genotype can be inferred with a *maximum a posteriori* (MAP) procedure.

Fetal genotype prior. In this context, the prior on fetal genotype $P(G)$ can be simply determined by Mendelian law based on genotypes of both parents (see appendix section S2.A2.1 for more details).

Data likelihood. At a given locus, the data likelihood $P(\text{data}|G)$ from Hoobari framework can be extended to our setting:

$$P(\text{data}|G) = \prod_{j=1}^{n} P(r_j|G, G_M, f) \tag{S2.2}$$

where $r_j \in \{0, 1\}$ is the allele carried by read j, and $j = 1, \dots, n$ indexes all reads covering the considered locus in the cfDNA sample, $G \in \{\,0/0,\ 0/1,\ 1/0,\ 1/1\,\}$ is the fetal genotype (with our specific fetal genotype encoding convention, i.e. $0/1 \neq 1/0$) and $G_M \in \{\,0/0,\ 0/1,\ 1/1\,\}$ is the maternal genotype (with the standard genotype encoding convention, i.e. $0/1 = 1/0$), and f the fetal fraction.

More details about read data likelihood computations are given in appendix section S2.A2.2.

S2.1.4.3 Fetal allele origin inference

We introduce an innovative approach to infer which allele was given to the fetus by each parent at the locus scale. The purpose is to identify, for a given genomic region, from which parental chromosome (among the pair of corresponding homologous chromosomes) originates the genetic material inherited by the fetus without a proband.

Notations. We introduce the following notation: for a given locus with fetal genotype A/B, the fetal allele origin will be noted X–Y where

$$X = \begin{cases} \texttt{mat1} & \text{if } A \text{ is the 1st maternal haplotype} \\ \texttt{mat2} & \text{if } A \text{ is the 2nd maternal haplotype} \end{cases} \tag{S2.3}$$

$$Y = \begin{cases} \texttt{pat1} & \text{if } B \text{ is the 1st paternal haplotype} \\ \texttt{pat2} & \text{if } B \text{ is the 2nd paternal haplotype} \end{cases} \tag{S2.4}$$

Then, the objective is to infer

$$X\text{–}Y \in \{\,\texttt{mat1–pat1},\ \texttt{mat1–pat2},\ \texttt{mat2–pat1},\ \texttt{mat2–pat2}\,\} \tag{S2.5}$$

for all locus of a genomic region (on a given chromosome). On contrary to the fetal genotype inference, we cannot consider each locus independently. Indeed, for a given parent, the inherited alleles for all locus in a contiguous region are likely to come from the same chromosome. Only a recombination event (unlikely between closed locus) or a phasing error (whose risk can be estimated) would make it possible for two consecutive SNPs to originate from different haplotypes. Hence, unless there was a recombination event or a phasing error between two consecutive SNPs (whose probability depends on their genetic distance and can be accounted for in the model), the fetal

allele origin will be the same for the two consecutive SNPs.

Working with multiple SNPs in a chromosomic region, the fetal allele origin at SNP/locus ℓ is noted

$$O_\ell = X\text{–}Y \in \{\,\texttt{mat1–pat1},\ \texttt{mat1–pat2},\ \texttt{mat2–pat1},\ \texttt{mat2–pat2}\,\} \quad \text{(S2.6)}$$

Then, the fetal allele origin over the complete region of multiple SNPs/locus is noted

$$O = \{O_1, \ldots, O_L\} = \{O_\ell\}_{\ell=1,\ldots,L} \quad \text{(S2.7)}$$

where L is the number of detected SNPs in the targeted region.

Inference. The full posterior

$$P(O \,|\, \text{data}) = P(O_1, \ldots, O_\ell, \ldots, O_L \,|\, \text{data}) \quad \text{(S2.8)}$$

gives the most probable fetal allele origin over all SNPs/locus in the considered region. Since consecutive SNPs are not independent, O_ℓ is not independent from $O_{\ell-1}$, and we cannot directly compute the full posterior as the product of marginal posteriors at the locus scale (like we did for the fetal genotype inference), i.e.

$$P(O_1, \ldots, O_\ell, \ldots, O_L \,|\, \text{data}) \neq \prod_{\ell=1}^{L} P(O_\ell \,|\, \text{data}).$$

Because of the combinatorial cost, computing the full posterior would be prohibitive, thus we need a workaround to infer it. To do so, we use a Gibbs sampler (S. Geman and D. Geman, 1984). In particular, we can use

$$P(O_\ell|\text{data}, O_{\ell-1}) \sim P(\text{data at locus } \ell|O_\ell) \times P(O_\ell|O_{\ell-1}) \quad \text{(S2.9)}$$

to simulate data under the full posterior (S2.8), which is discrete with a finite size state space. Then, by sampling enough points, we can estimate the locus posterior $P(O_\ell|\text{data})$ and the fetal allele origin over the targeted region.

It can be noted that the data likelihood $P(\text{data at locus } \ell|O_\ell)$ can be explicitly derived as in the fetal genotype model (c.f. section S2.1.4.2) and the transition probability $P(O_\ell|O_{\ell-1})$ between locus ℓ and $\ell-1$ can be computed depending on the probability of a recombination event between locus ℓ and $\ell - 1$ in each parent (which depends on the genetic distance between the

consecutive locus), and the probability of a phasing error at locus ℓ (which is estimated by the phasing software).

More details regarding Gibbs sampling and our Bayesian approach can be found in appendix section S2.A3.

Appendix

S2.A1 Fetal fraction estimation

Here is a detailed explanation of the approach from Lo et al. (2010) and Chan and Jiang (2015) to estimate the fetal fraction at locus level using the number of reads that map to a SNP locus in the cfDNA sample.

We introduce the following notations to decompose the number of reads that map at a given locus in the cfDNA sample:

- N_{total} = total number of reads
- N_{fetus} = number of reads from the fetus
- N_{mother} = number of reads from the mother
- f = fetal fraction (or $\mathcal{F}$ with percentage notation, i.e. $\mathcal{F} = (100 \times f)\,\%$)

By definition of the fetal fraction, we have the following link between N_{fetus} and N_{mother}:

$$N_{fetus} = f \times N_{total} \tag{S2.A10}$$

$$N_{mother} = (1 - f) \times N_{total} \tag{S2.A11}$$

$$f = \frac{N_{fetus}}{N_{total}} \tag{S2.A12}$$

The genotypes will be noted as follow (for any given alleles[1] "A" and "C"): "A/A" or "C/C" for homozygous locus, and "A/C" or "C/A" for heterozygous locus.

In the following, we will use these notations for the allelic depths at a given locus in the cfDNA sample:

- N_A = number of reads with allele "A"
- N_C = number of reads with allele "C"

The total number of reads at the considered locus is then:

$$N_{total} = N_A + N_C \tag{S2.A13}$$

[1]Here "A" and "C" are just blind notations for the different alleles carried by a given locus, among "0" for the ancestral allele, and "1" for the derived/mutated allele. They are not referring to sequenced DNA nucleotids.

S2.A1.1 Local fetal fraction

The Table S2.A1 recapitulates the different cases for the expected allelic depth at a given locus, depending on the maternal and paternal genotypes, the cfDNA sample genotype and the corresponding unknown fetal genotype.

Based on relations defined in Table S2.A1, the Table S2.A2 details the corresponding estimate (noted $\widehat{f}$) for the fetal fraction at the given locus.

Mother	Father	cfDNA	Fetus[a]	Expected proportions for allelic depth[b]	
A/A	A/A	A/A	A/A	100% of reads "A"	
A/A	C/C	A/C	A/C	$(100 - \mathcal{F})\,\%$ of reads "A" and $\mathcal{F}\,\%$ of reads "C"	(I)
A/A	A/C	A/C	A/C	$(100 - 0.5\,\mathcal{F})\,\%$ of reads "A" and $(0.5\,\mathcal{F})\,\%$ of reads "C"[c]	(II)
		A/A	A/A	100% of reads "A"	
A/C	A/A	A/C	A/C	50% of reads "A" and 50% of reads "C"	(III)
			A/A	$(50 + 0.5\,\mathcal{F})\,\%$ "A" and $(50 - 0.5\,\mathcal{F})\,\%$ "C"[d]	
A/C	A/C	A/C	A/C	50% of reads "A" and 50% of reads "C"	(IV)
			A/C	50% of reads "A" and 50% of reads "C"	
			A/A	$(50 + \mathcal{F})\,\%$ of reads "A" and $(50 - \mathcal{F})\,\%$ of reads "C"[e]	
			C/C	$(50 - \mathcal{F})\,\%$ of reads "A" and $(50 + \mathcal{F})\,\%$ of reads "C"[f]	

Table S2.A1: Expected allele proportions at a given locus depending on maternal, paternal, cfDNA and (unknown) fetal genotypes (Lo et al., 2010; Chan and Jiang, 2015), where $\mathcal{F} = (100 \times f)\,\%$ is the percentage notation for the fetal fraction f.

[a] unobserved
[b] in the cfDNA sample at the considered locus
[c] i.e. $N_{total} = (1 - 0.5\,f)\,N_A + 0.5\,f\,N_C$
[d] i.e. $N_{total} = (0.5 + 0.5\,f)\,N_A + (0.5 - 0.5\,f)\,N_C$
[e] i.e. $N_{total} = (0.5 + f)\,N_A + (0.5 - f)\,N_C$
[f] i.e. $N_{total} = (0.5 - f)\,N_A + (0.5 + f)\,N_C$

Mother	Father	cfDNA	Fetus[a]	Estimated fetal fraction[b]	
A/A	A/A	A/A	A/A	×	
A/A	C/C	A/C	A/C	$\widehat{f} = 2 \times N_C/N_{total}$	(I)
A/A	A/C	A/C	A/C	$\widehat{f} = 2 \times N_C/N_{total}$	(II)
		A/A	A/A	×	
A/C	A/A	A/C	A/C	×	(III)
			A/A	$\widehat{f} = (N_A - N_C)/N_{total}$	
A/C	A/C	A/C	A/C	×	(IV)
			A/C	×	
			A/A	$\widehat{f} = (N_A - N_C)/N_{total}$	
			C/C	$\widehat{f} = (N_C - N_A)/N_{total}$	

Table S2.A2: Estimated local fetal fraction at a given locus depending on maternal, paternal, cfDNA and (unknown) fetal genotypes (Lo et al., 2010; Chan and Jiang, 2015), where $\widehat{f}$ is the estimated fetal fraction. × identifies cases where the fetal fraction cannot be estimated.

[a]unobserved
[b]in the cfDNA sample at the considered locus

From Tables S2.A1 and S2.A2, when the cfDNA genotype is "A/C" or "C/A" (i.e. when the mother is heterozygous and the father is either homozygous or heterozygous, corresponding to cases III and IV respectively), to be able to estimate the local fetal fraction, we need to discriminate between different scenarios, corresponding to uninformative situations regarding the fetal fraction (where the allelic depths are expected to exactly be 50% of allele A and 50% of allele C independently from the fetal fraction, i.e. $N_A = N_C$) versus other informative situations where the fetal fraction can be estimated[2]. This discrimination depends on the fetal genotype which is unknown. Therefore, the only condition that we can check to discriminate between informative and uninformative situations is whether $N_A = N_C$ or not.

In practice, the situation of perfectly balanced allelic depths, i.e. $N_A = N_C$, is very unlikely to happen, because of sequencing technical variability. Thus, at a given locus with genotype "A/C" in the cfDNA sample, if we have $N_A \approx N_C$, it is difficult to determine whether it corresponds to the uninformative (regarding the fetal fraction estimation) situation where $N_A = N_C$, or to one of the other informative situations when the fetal fraction f is very small (i.e. $|N_A - N_C|$ close to 0).

In practice, we use the following convention. Cases I and II are not ambiguous and can always be used. Cases III and IV are ambiguous (because the fetal genotype is unknown), thus locus where $N_A \approx N_C$ are discarded because it is not possible to discriminate between the two case: "$N_A = N_C$" or "very small fetal fraction". To do so, we check the following conditions

$$\frac{|N_A - N_C|}{N_{total}} > \text{tol}$$

at any given locus, for a given tolerance threshold tol (we used 5%). One limit is that it is not possible to estimate the fetal fraction when it is very small with this approach.

In addition the conditions $N_A > N_C$ or $N_C > N_A$ is used to infer which scenario should be used in case IV.

[2] i.e. $\widehat{f} = \frac{N_A - N_C}{N_{total}}$ or $\frac{N_C - N_A}{N_{total}}$, c.f. Table S2.A2.

mat \ pat	0/0	0/1	1/1
0/0	P(0/0) = 1	P(0/0) = 0.5<br>P(0/1) = 0.5	P(0/1) = 1
0/1	P(0/0) = 0.5<br>P(1/0) = 0.5	P(0/0) = 0.25<br>P(0/1) = 0.25<br>P(1/0) = 0.25<br>P(1/1) = 0.25	P(0/1) = 0.5<br>P(1/1) = 0.5
1/1	P(1/0) = 1	P(1/0) = 0.5<br>P(1/1) = 0.5	P(1/1) = 1

Table S2.A3: Prior proabalities on fetal genotype depending on maternal (noted "mat") and paternal ("pat") genotypes for a given locus according to Mendelian inheritance law.

S2.A1.2 Smoothing

To smooth the local estimations, and to get an estimation of the fetal fraction at locus where it was not possible to derive an estimate (c.f. previous section), we use a window-based averaging of local fetal fraction estimations, with a 100kb-wide window around each SNP. It should be noted that when not enough local estimates can be found in the window, a chromosome-wide averaging is used.

S2.A2 Fetal genotype model

S2.A2.1 Fetal genotype prior

The Table S2.A3 summarizes the Mendelian law of genotype inheritance (c.f. Bateson and Mendel, 2009) for a given locus using the specific fetal genotype notation introduced earlier.

S2.A2.2 Data likelihood

The data likelihood, i.e. the likelihood for all reads covering the considered locus in the cfDNA sample, can be explicitly computed. Assum-

ing[3] $G_M \in \{\,0/0,\ 0/1,\ 1/1\,\}$ is the observed maternal genotype, and $G \in \{\,0/0,\ 0/1,\ 1/0,\ 1/1\,\}$ is a fetal genotype compatible with the observed data (i.e. compatible with the maternal genotype), the likelihood for read r_j can be written (Rabinowitz et al., 2019):

$$\begin{aligned} &P(r_j = x \,|\, G = fet, G_M, f) \\ &\qquad = P(r_j = x \,|\, G)\, P(G \,|\, f) + P(r_j = x \,|\, G_M)\, P(G_M \,|\, f) \end{aligned} \tag{S2.A14}$$

where x is the allele carried by read j, and f the fetal fraction.

It should be noted that Equation (S2.A14) is a notation simplification not strictly rigorous, where the dependence between G and G_M is implicit and hidden.

Based on Equation (S2.A14), it is possible to compute $P(r_j = x \,|\, G = fet, G_M, f)$ for $x = 0$ and $x = 1$, see Tables S2.A4 and S2.A5 respectively.

[3] using standard genotype notation for the mother, and our specific genotype convention for the fetus.

fet / mat	0/0	0/1	1/0	1/1
0/0	1	$0.5 \times f + (1 - f)$	$\times$	$\times$
0/1	$f + 0.5 \times (1 - f)$	$0.5 \times f + 0.5 \times (1 - f)$	$0.5 \times f + 0.5 \times (1 - f)$	$0.5 \times (1 - f)$
1/1	$\times$	$\times$	$0.5 \times f + (1 - f)$	0

Table S2.A4: Detailed computation of $P(r_j = 0 \,|\, G = \text{“fet”}, G_M = \text{“mat”}, f)$ for observed maternal genotype “mat” and compatible fetal genotype. $\times$ identifies impossible cases (conflicting maternal and fetal genotypes).

fet / mat	0/0	0/1	1/0	1/1
0/0	0	$0.5 \times f$	$\times$	$\times$
0/1	$0.5 \times (1 - f)$	$0.5 \times f + 0.5 \times (1 - f)$	$0.5 \times f + 0.5 \times (1 - f)$	$0.5 \times (1 - f)$
1/1	$\times$	$\times$	$0.5 \times f + (1 - f)$	1

Table S2.A5: Detailed computation of $P(r_j = 1 \,|\, G = \text{“fet”}, G_M = \text{“mat”}, f)$ for observed maternal genotype “mat” and compatible fetal genotype. $\times$ identifies impossible cases (conflicting maternal and fetal genotypes).

S2.A3 Fetal allele origin inference

Recalling that the fetal allele origin at SNP/locus ℓ is noted

$$O_\ell = X\text{–}Y \in \{\,\texttt{mat1–pat1},\ \texttt{mat1–pat2},\ \texttt{mat2–pat1},\ \texttt{mat2–pat2}\,\},$$

the fetal allele origin over the complete region of multiple SNPs/locus is noted

$$O = \{O_1, \dots, O_L\} = \{O_\ell\}_{\ell=1,\dots,L}$$

where L is the number of detected SNPs in the targeted region.

Gibbs sampler. Because of dependency between consecutive locus, we have

$$P(O \,|\, \text{data}) = P(O_1, \dots, O_\ell, \dots, O_L \,|\, \text{data}) \neq \prod_{\ell=1}^{L} P(O_\ell \,|\, \text{data}), \quad \text{(S2.A15)}$$

and in particular since the data likelihood cannot be factorized:

$$P(\text{data} \,|\, O) = P(\text{data} \,|\, O_1, \dots, O_\ell, \dots, O_L) \neq \prod_{\ell=1}^{L} P(\text{data} \,|\, O_\ell). \quad \text{(S2.A16)}$$

Thanks to a Gibbs sampling procedure (c.f. appendix section S2.A3.1), successive samplings under conditional posterior

$$P(O_\ell|\text{data}, O_1, \dots, O_{\ell-1}, O_{\ell+1}, \dots, O_L) \quad \text{(S2.A17)}$$

can be used to approximate a sampling under the joint posterior

$$P(O_1, \dots, O_\ell, \dots, O_L|\text{data})$$

In our specific context, the conditional posterior (S2.A17) can be simplified as

$$P(O_\ell|\text{data}, O_1, \dots, O_{\ell-1}, O_{\ell+1}, \dots, O_L) = P(O_\ell|\text{data}, O_{\ell-1}) \quad \text{(S2.A18)}$$

because the allele origin O_ℓ at locus ℓ does only depend on the allele origin $O_{\ell-1}$ at locus $\ell - 1$ (c.f. Ghahramani, 2001). The simplified conditional posterior (S2.A18) is then explicit:

$$P(O_\ell|\text{data}, O_{\ell-1}) \sim P(\text{data at locus } \ell|O_\ell) \times P(O_\ell|O_{\ell-1}) \quad \text{(S2.A19)}$$

Decomposing the simplified conditional posterior (S2.A19), we have:

– the *data likelihood*

$$P(\text{data at locus } \ell | O_\ell, O_{\ell-1}) = \prod_j P(r_j | O_\ell)$$

with r_j = allele of read j where j indexes all reads covering locus ℓ.

– the *transition probability* $P(O_\ell | O_{\ell-1})$ depending on the recombination probability between locus $\ell - 1$ and ℓ and the phasing error likelihood at locus ℓ

Data likelihood. The data likelihood is decomposed as follows

$$\prod_j P(r_j | O_\ell) = \prod_j \sum_{g \in \mathcal{G}_F} P(r_j | G = g, H_M, f) \times P(G = g | H_M, H_F, f, O_\ell) \tag{S2.A20}$$

where

– $r_j \in \{0, 1\}$ is the allele carried by read j at locus ℓ

– $\mathcal{G}_F = \{\, 0/0,\ 0/1,\ 1/0,\ 1/1 \,\}$ is the set of possible fetal genotypes at locus ℓ (using our our specific fetal genotype encoding convention)

– $G \in \mathcal{G}_F$ is the unknown fetal genotype at locus ℓ

– $H_M \in \{\, 0|0,\ 0|1,\ 1|0,\ 1|1 \,\}$ is the mother haplotype at locus ℓ

– $H_F \in \{\, 0|0,\ 0|1,\ 1|0,\ 1|1 \,\}$ is the father haplotype at locus ℓ

– f is the fetal fraction fetal fraction

The *read likelihood* $P(r_j | G, H_M, f)$ can be computed as previously in the fetal genotype model, c.f. equation (S2.A14) in appendix section S2.A2.2, and the *prior* $P(G | G_M, G_F, f, O_\ell)$ can be deduced from the Mendelian law (c.f. appendix section S2.A2.1).

Transition probabilities. The transition probability $P(O_\ell | O_{\ell-1})$ between locus ℓ and locus $\ell - 1$ depends on:

- the *recombination probability* between locus $\ell - 1$ and ℓ in each parent:

$$r_\ell = \texttt{dist} \times \rho$$

where `dist` is the distance (in bp) between locus ℓ and $\ell - 1$, and ρ is the recombination rate

- the *phasing error probability* at locus ℓ in each parent, noted e_ℓ^M for the mother and e_ℓ^F for the father (c.f. `PQ` and `JQ` fields in 10x VCF files)

Thus, the *switch probability* for the mother corresponds to the probability that locus ℓ was not inherited from the same haplotype as locus $\ell-1$, i.e. the probability of the following events between locus ℓ and $\ell-1$: "recombination AND no phasing error" OR "no recombination AND phasing error":

$$\begin{aligned} p_\ell^M &= P(O_\ell = X_1\text{–}Y_1 | O_{\ell-1} = X_2\text{–}Y_2 \text{ and } X_1 \neq X_2) \\ &= r_\ell \, (1 - e_\ell^M) + (1 - r_\ell) \, e_\ell^M \end{aligned} \tag{S2.A21}$$

where $X_1\text{–}Y_1, X_2\text{–}Y_2 \in \{\texttt{mat1–pat1}, \texttt{mat1–pat2}, \texttt{mat2–pat1}, \texttt{mat2–pat2}\}$. Similarly, the switch probability for the father is

$$p_\ell^F = r_\ell \, (1 - e_\ell^F) + (1 - r_\ell) \, e_\ell^F \tag{S2.A22}$$

By combining equations (S2.A21) and (S2.A22), we can compute the transition probability $P(O_\ell | O_{\ell-1})$ as detailed in Figure S2.A1.

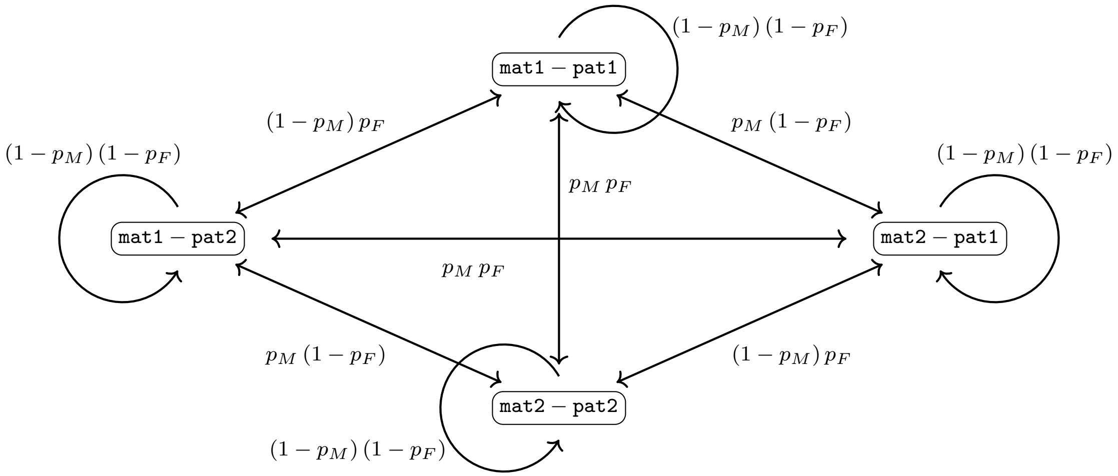

Figure S2.A1: Computing $P(O_\ell | O_{\ell-1})$. The arrow indicates the transition from $O_{\ell-1}$ to O_ℓ with the corresponding probabilities. Here, p^M refers to the switch probability p_ℓ^M for the mother at locus ℓ, c.f. equation (S2.A21), and p^F refers to the switch probability p_ℓ^F for the father at locus ℓ, c.f. equation (S2.A22).

Pipeline. The pipeline to infer the fetal allele origin can be summarized as follow:

1. Initialize fetal allele origins $\{O_1, \ldots, O_\ell, \ldots, O_L\}$ (thanks to a heuristic based on non-ambiguous locus)

2. Parallel sampling under posterior $P(O_1, \ldots, O_\ell, \ldots, O_L|data)$ with the Gibbs sampler

3. From the previous samples, estimation of the marginal posterior $P(O_\ell|data)$

4. Infer $O_\ell = X\text{–}Y \in \{$ `mat1–pat1`, `mat1–pat2`, `mat2–pat1`, `mat2–pat2` $\}$ with marginal MAP

S2.A3.1 Reminder about Gibbs sampling

See Yildirim (2012) for an introduction about Gibbs sampling for Bayesian inference.

Assuming we are working with $X_1, \ldots, X_n$ a set of n random variables of unknown joint distribution $P(X_1, \ldots, X_n)$, we construct a sequence $\{\mathbf{X}^{(t)}\}_{t\geq 1}$ of simulated values where $\mathbf{X}^{(t)} = \left(x_1^{(t)}, \ldots, x_n^{(t)}\right)$ with the algorithm 1.

Algorithm 1: Gibbs sampler algorithm

Initialization with some given values $\mathbf{X}^{(0)} = \left(x_1^{(0)}, \ldots, x_n^{(0)}\right)$;
for $t \leftarrow 1, 2$ **to** l **do**
  for $i \leftarrow 1$ **to** n **do**
    Sample a value $x_i^{(t+1)}$ from the conditional distribution

$$P\Big(X_i \Big| X_1 = x_1^{(t+1)}, \ldots, X_{i-1} = x_{i-1}^{(t+1)}, X_{i+1} = x_{i+1}^{(t)}, X_n = x_n^{(t)}\Big)$$

  end
end

It can be shown that, after enough iterations over t, $\left(x_1^{(t)}, \ldots, x_n^{(t)}\right)$ is sampled under unknown joint distribution $P(X_1, \ldots, X_n)$. In practice, to get numerous samples under $P(X_1, \ldots, X_n)$, it is recommended to run a burn-in period until $t > T$, an then to keep the sample $\mathbf{X}^{(t)}$ every τ iterations. The lag τ is used to avoid correlations between the samples, it can be calibrated

based on auto-correlation. In our computations, we used $T = 2000$ for the burn-in period, $\tau = 100$ for the lag, and we computed 5000 samples.

References

Andrieu, Christophe et al. (Jan. 2003). "An Introduction to MCMC for Machine Learning". en. In: *Machine Learning* 50.1, pp. 5–43. ISSN: 1573-0565. DOI: 10.1023/A:1020281327116.

Bateson, William and Gregor Mendel (2009). *Mendel's Principles of Heredity: A Defence, with a Translation of Mendel's Original Papers on Hybridisation*. Cambridge Library Collection - Darwin, Evolution and Genetics. Cambridge: Cambridge University Press. ISBN: 978-1-108-00613-2. DOI: 10.1017/CBO9780511694462.

Bolstad, William M. and James M. Curran (2016). *Introduction to Bayesian statistics*. John Wiley & Sons.

Chan, Landon L. and Peiyong Jiang (Oct. 2015). "Bioinformatics analysis of circulating cell-free DNA sequencing data". eng. In: *Clinical Biochemistry* 48.15, pp. 962–975. ISSN: 1873-2933. DOI: 10.1016/j.clinbiochem.2015.04.022.

Choi, Yongwook et al. (Apr. 2018). "Comparison of phasing strategies for whole human genomes". In: *PLoS Genetics* 14.4. ISSN: 1553-7390. DOI: 10.1371/journal.pgen.1007308.

Geman, Stuart and Donald Geman (Nov. 1984). "Stochastic Relaxation, Gibbs Distributions, and the Bayesian Restoration of Images". In: *IEEE Transactions on Pattern Analysis and Machine Intelligence* PAMI-6.6. Conference Name: IEEE Transactions on Pattern Analysis and Machine Intelligence, pp. 721–741. ISSN: 1939-3539. DOI: 10.1109/TPAMI.1984.4767596.

Ghahramani, Zoubin (Feb. 2001). "An introduction to hidden markov models and bayesian networks". In: *International Journal of Pattern Recognition and Artificial Intelligence* 15.01. Publisher: World Scientific Publishing Co., pp. 9–42. ISSN: 0218-0014. DOI: 10.1142/S0218001401000836.

Huang, Mengting, Jing Tu, and Zuhong Lu (Sept. 2017). "Recent Advances in Experimental Whole Genome Haplotyping Methods". In: *International Journal of Molecular Sciences* 18.9. ISSN: 1422-0067. DOI: 10.3390/ijms18091944.

Hui, Lisa and Diana W. Bianchi (2020). "Fetal fraction and noninvasive prenatal testing: What clinicians need to know". en. In: *Prenatal Diagnosis* 40.2, pp. 155–163. ISSN: 1097-0223. DOI: 10.1002/pd.5620.

Lo, Y. M. Dennis et al. (Dec. 2010). “Maternal plasma DNA sequencing reveals the genome-wide genetic and mutational profile of the fetus”. eng. In: *Science Translational Medicine* 2.61, 61ra91. ISSN: 1946-6242. DOI: 10.1126/scitranslmed.3001720.

Peng, Xianlu Laura and Peiyong Jiang (Feb. 2017). “Bioinformatics Approaches for Fetal DNA Fraction Estimation in Noninvasive Prenatal Testing”. In: *International Journal of Molecular Sciences* 18.2. ISSN: 1422-0067. DOI: 10.3390/ijms18020453.

Rabinowitz, Tom et al. (Mar. 2019). “Bayesian-based noninvasive prenatal diagnosis of single-gene disorders”. In: *Genome Research* 29.3, pp. 428–438. ISSN: 1088-9051. DOI: 10.1101/gr.235796.118.

Yildirim, Ilker (2012). “Bayesian inference: Gibbs sampling”. In: *Technical Note, University of Rochester*.
