# Supplementary material for: Noninvasive prenatal diagnosis of genetic diseases induced by triplet repeat expansion by linked read haplotyping and Bayesian approach
Source: Sci Rep. 2022 Jul 6;12:11423. doi: 10.1038/s41598-022-15307-2 (PMC9259573; doi:10.1038/s41598-022-15307-2)
Supplement: Supplementary file 3 — Supplementary Information 3. [file 41598_2022_15307_MOESM3_ESM.pdf]

Non-invasive prenatal diagnostic of genetic diseases induced by triplet repeats expansion by linked reads haplotyping and Bayesian approach

Supplementary materials 3: additional statistical analyses

June 14, 2022

Liautard-Haag C.[1], Durif G.[2], Van Goethem C.[1,3], Baux D.[1,4], Louis A.[1], Cayrefourcq L.[5], Lamairia M.[1], Willems M.[6], Zordan C.[7], Dorian V.[7], Rooryck C.[7], Goizet C.[8], Chaussenot A.[8], Monteil L.[9], Calvas P.[9], Miry C.[10], Favre R.[10], Le Boette E.[11], Fradin M.[11], Roux AF.[1,4], Cossée M.[1,3], Koenig M.[1,3], Panabière C.[3], Guissart C.[1], Vincent MC.[1,3]

[1] Laboratoire de Génétique Moléculaire, Institut Universitaire de Recherche Clinique, Université de Montpellier, CHU Montpellier, Montpellier, France;
[2] IMAG, Université de Montpellier, CNRS, Montpellier, France;
[3] PhyMedExp Univ. Montpellier, CNRS, INSERM, Montpellier, France;
[4] INM, Institut des neurosciences de Montpellier, INSERM U1298, Montpellier, France;
[5] Laboratory of Rare Human Circulating Cells (LCCRH), University Medical Center of Montpellier, Montpellier, France;
[6] Département de Génétique Médicale, Maladies Rares et Médecine Personnalisée, Centre de Référence Anomalies du Développement et Syndromes Malformatifs, Université de Montpellier, CHU de Montpellier, France;
[7] Service de Génétique Médicale, Groupe Hospitalier Pellegrin, CHU de Bordeaux, Bordeaux, France;
[8] Service de Génétique Médicale, Centre de Référence des Maladies Mitochondriales, Hôpital de l'Archet 2, Nice, France;
[9] Service de Génétique Médicale, CHU de Toulouse, Toulouse, France;
[10] Department of Maternal Fetal Medicine, Strasbourg University Hospital, Strasbourg, France;
[11] Service de Génétique Médicale, Centre Hospitalier de Saint Brieuc, Saint-

Brieuc, France.

Contents

S3.1 Additional statistical analyses **3**
S3.1.1 Sequencing data summary 3
S3.1.2 Quantitative and qualitative analyses 4
S3.1.3 Size of phased region 4
S3.1.4 Result of Bayesian inference 4

S3.1 Additional statistical analyses

In this section, we present a few statistical investigations about the sequencing data in our study, in particular to highlight possible explanation about (*i*) parental genotype phasing result (which are variable depending on the subjects), and (*ii*) about the outcome of our NIPD (Non-Invasive Prenatal Diagnosis procedure) approach (which is conclusive[1] for some family, and non conclusive for other).

S3.1.1 Sequencing data summary

In order to find preliminary answers and possible hints explaining our results, we focus on the following quantities (quantitative variables or features) that were measured/recorded regarding sequencing data from all parents in the study (including 12 families, i.e. 24 parents):

- nb. of sequencing reads: number of reads collected by the sequencing
- target depth: average coverage on the target region
- nb. of SNPs: number of SNPs in the 200Kbp target region
- mean depth: global average sequencing coverage
- prop. of large DNA: proportion of DNA fragment longer than 20Kbp
- size of phased region: length of the phased region around the expansion
- DNA integraty value: DNA quality control quantifier
- size peak position: position of the peak of DNA fragment size distribution (in bp)

It should be noted that "size peak position" and "DNA integraty value" were missing for two parents of the same family.
In addition, we also considered the following qualitative factors:

- DNA size selection: indicator ("yes"/"no") about specific procedure to eliminate short DNA reads (used when the proportion of long DNA fragments is too small)

[1]Here "conclusive" means that the algorithm was able to infer a result with a certain level of certainty. On the opposite, "non-conclusive" means that the uncertainty on the algorithm inference was too large and therefore the result not trustworthy. This uncertainty is directly estimated by the Bayesian procedure, through the posterior distribution estimation.

– NIPD outcome: indicator ("yes"/"no") about the Bayesian procedure result determination ("conclusive", including "affected" or "not affected" versus "non conclusive")

– subject: indicator about parent individual ("mother" or "father")

– affected: indicator about affected/carrier parent ("yes"/"no")

S3.1.2 Quantitative and qualitative analyses

Figure S3.1 presents the density plot for all quantitative variables, and pairwise bootstrap correlations between aforementioned variables. We use bootstrap correlation to validate the correlation measure in relation with the small sample size. The objective was to highlight potential relations between the different features characterizing our sequencing data.

Table S3.1 presents the result about the non-parametric Mann-Withney U test (Mann and Whitney, 1947), also knows as Mann–Whitney–Wilcoxon test, regarding distribution equality in two populations. Here, we considered the various qualitative factors to separate observations of the different quantitative variables (previously described, c.f. subsection S3.1.1). We used a non-parametric test to account for the small sample size, with the purpose to unravel potential link the quantitative features and the qualitative factors.

S3.1.3 Size of phased region

According to figure S3.1, there is a potential link (positive correlation > 0.6) between the size of the phased region and the sequencing depth on the target region on one side, and the proportion of large DNA on the other side. This suggest that a high coverage sequencing of long DNA reads could have a positive effect on phasing result. This point should be further investigated in a future study.

S3.1.4 Result of Bayesian inference

Table S3.1 suggests that differences in the sequencing depth in target region or in the size of the phased region are associated with the outcome of the NIPD procedure.

Figures S3.2 and S3.3 (which are restricted to affected/carrier parents) illustrate this potential link. It appears that conclusive results are obtained

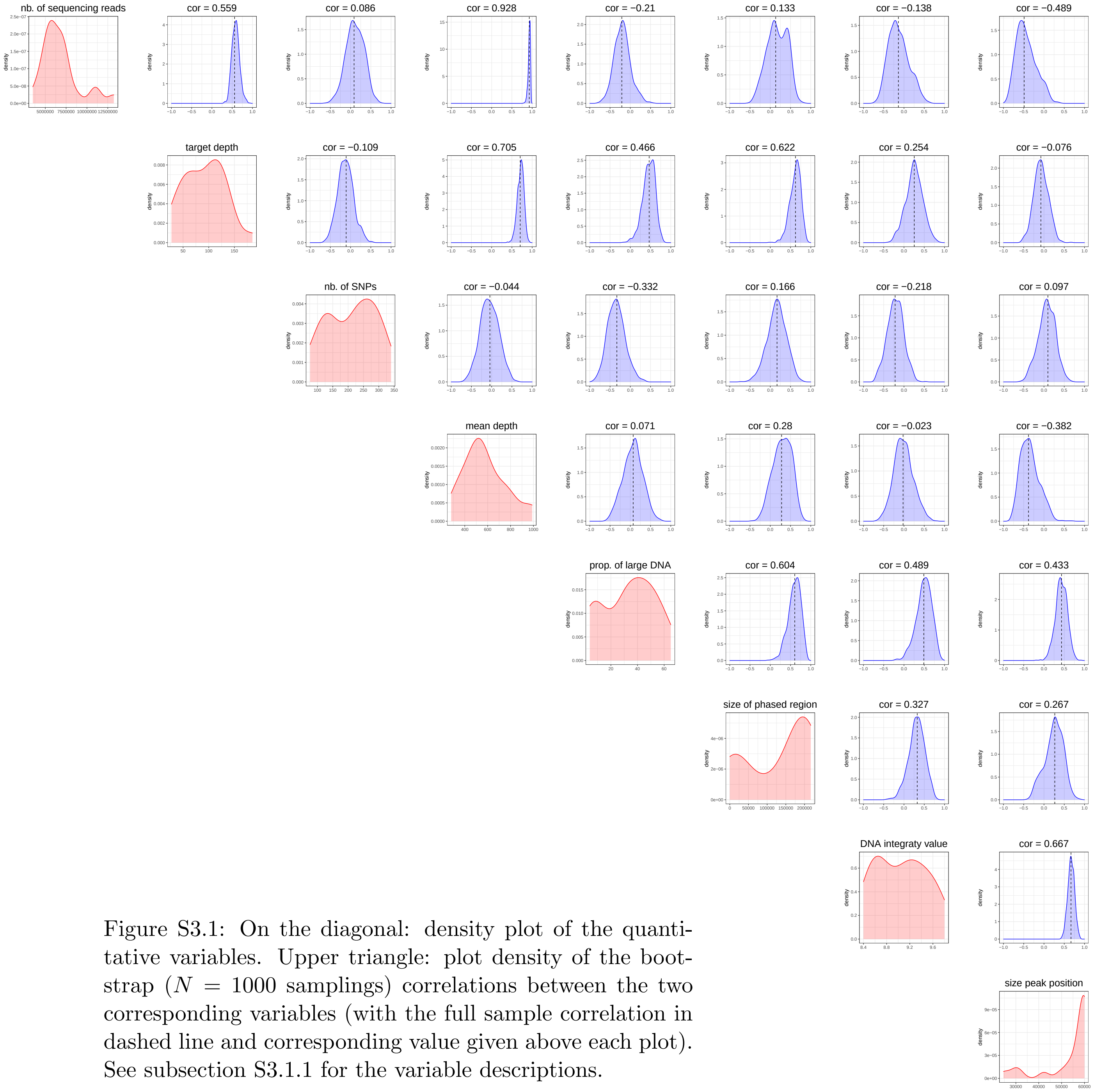

Figure S3.1: On the diagonal: density plot of the quantitative variables. Upper triangle: plot density of the bootstrap ($N = 1000$ samplings) correlations between the two corresponding variables (with the full sample correlation in dashed line and corresponding value given above each plot). See subsection S3.1.1 for the variable descriptions.

with parents where the size of the phased region and the target depth are higher. Therefore, the performance of our approach seems to be sensitive to the sequencing quality and to the parental genotype phasing quality.

Quant. var.	Qualit. factor	p-value
nb. of sequencing reads	DNA size selection	0.12280
target depth	DNA size selection	0.71798
nb. of SNPs	DNA size selection	0.07615
mean depth	DNA size selection	0.71798
prop. of large DNA	DNA size selection	0.01975
size of phased region	DNA size selection	0.57652
DNA integraty value	DNA size selection	0.01105
size peak position	DNA size selection	0.00878
nb. of sequencing reads	NIPD outcome	0.13321
target depth	NIPD outcome	0.01401
nb. of SNPs	NIPD outcome	0.78479
mean depth	NIPD outcome	0.15177
prop. of large DNA	NIPD outcome	0.53904
size of phased region	NIPD outcome	0.07034
DNA integraty value	NIPD outcome	0.15962
size peak position	NIPD outcome	0.82975
nb. of sequencing reads	subject	0.45016
target depth	subject	0.30877
nb. of SNPs	subject	0.69351
mean depth	subject	0.13955
prop. of large DNA	subject	0.62237
size of phased region	subject	0.89546
DNA integraty value	subject	0.06460
size peak position	subject	0.33435
nb. of sequencing reads	affected	0.81823
target depth	affected	0.57674
nb. of SNPs	affected	0.18896
mean depth	affected	0.92154
prop. of large DNA	affected	0.15801
size of phased region	affected	0.74253
DNA integraty value	affected	0.92114
size peak position	affected	0.73026

Table S3.1: Result about Mann-Whitney U test for distribution equality for the different quantitative variables depending on the qualitative factor. See subsection S3.1.1 for the variable descriptions.

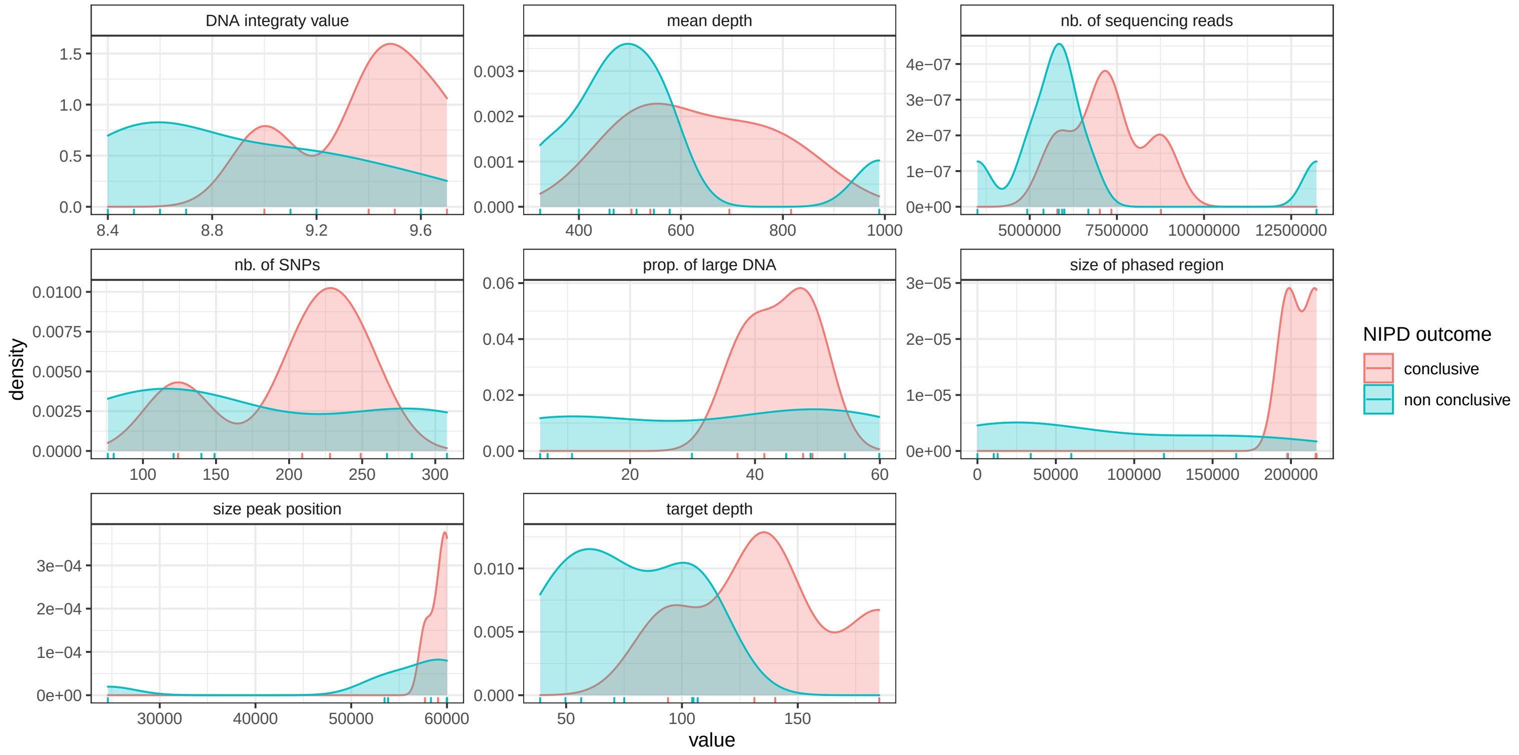

Figure S3.2: Density plot for the quantitative variables, for affected/carrier parents, discriminated by NIPD outcome indicator. See subsection S3.1.1 for the variable descriptions.

References

Mann, H. B. and D. R. Whitney (Mar. 1947). “On a Test of Whether one of Two Random Variables is Stochastically Larger than the Other”. In: *The Annals of Mathematical Statistics* 18.1. Publisher: Institute of Mathematical Statistics, pp. 50–60. ISSN: 0003-4851, 2168-8990. DOI: 10.1214/aoms/1177730491.

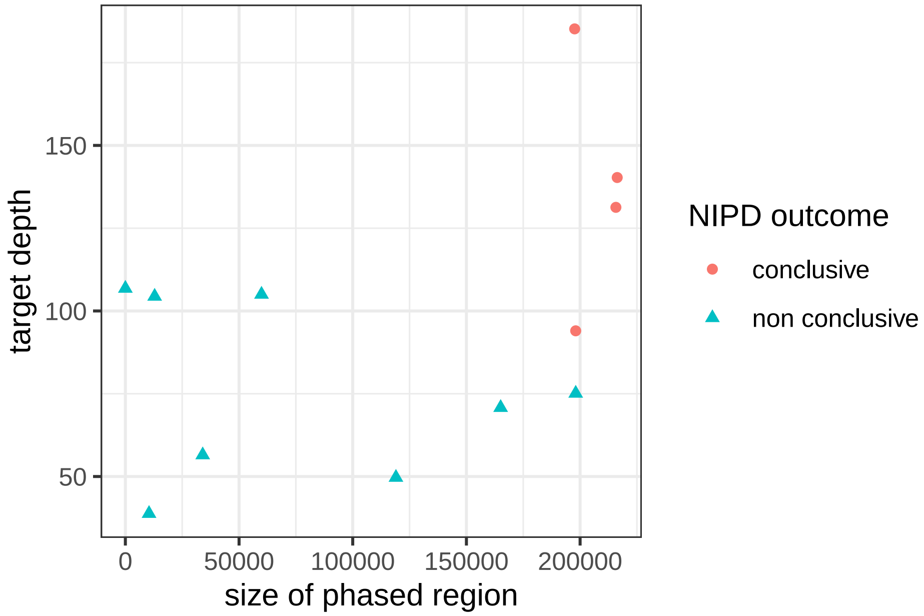

Figure S3.3: Target depth vs Size of phased region for affected/carrier parents, with NIPD outcome indicator. See subsection S3.1.1 for the variable descriptions.
